# Risk Factors for One-Year Post-Nephrectomy Decline in Renal Function of Living Kidney Donors: Quantile Regression Analysis Based on Estimated Glomerular Filtration Rate Reduction Percentiles

**DOI:** 10.3389/ti.2025.14749

**Published:** 2025-06-23

**Authors:** Alfonso Hernandez Santos, Hisham Ibrahim, Kawther Alquadan, Amer Belal, Muhannad Leghrouz, Rohan Mehta, Xuerong Wen, Georgios Vrakas

**Affiliations:** ^1^ Department of Medicine, College of Medicine, University of Florida, Gainesville, FL, United States; ^2^ College of Pharmacy, University of Rhode Island, Kingston, RI, United States; ^3^ Department of Surgery, College of Medicine, University of Florida, Gainesville, FL, United States

**Keywords:** quantile regression, donor nephrectomy, renal function reduction, risk factors, living kidney donor

Dear Editors,

The association of risk factors with renal outcomes of living kidney donors (LKDs) is typically analyzed with models using the average glomerular filtration rate (GFR) or its change over time [1]. Hence, risk factors associated with the decline of LKDs’ renal function beyond the average or central measure are not typically characterized. Therefore, there is a need to identify risk factors for higher-than-average severity of renal function deterioration post-donor nephrectomy (post-DN) in LKDs, considering the increasing trend of accepting medically complex LKD candidates [2, 3].

This study aimed to identify pre-donation demographic, anthropometric, and systemic blood pressure (BP)-related risk factors associated with the mean and the 50th, 75th, 90th, and 95th percentiles of eGFR reduction 1-year post-DN in LKDs and show the applicability of quantile regression (QR) in analyzing quantitative transplant data. Compared with ordinary least squares regression (OLSR), which concentrates on the average outcomes, QR can find how risk factors influence varying degrees or severities of outcomes. Additionally, QR is more robust to outliers and non-normal data than OLSR [4].

After institutional review board approval of the study protocol, we used the transplant center data to study 37 LKDs between 7/2012 and 12/2023 with complete baseline pre-kidney donation demographics, anthropometric, serum creatinine, and 12th-month post-DN blood pressure and serum creatinine records. We calculated the LKDs’ estimated GFR (eGFR) based on the Chronic Kidney Disease Epidemiology Collaboration (CKD-Epi) [5] formula, pre-DN, and 1-year post-DN. The study outcome was the percentage change in the LKDs’ CKD-Epi eGFR between pre- and 1-year post-DN. We analyzed associations between risk factors and the outcome using bivariate OLSR and bivariate quantile regressions (QRs) in the 50th, 75th, 90th, and 95th percentiles of eGFR reduction in percent at 1-year post-DN [4]. When applicable, we performed multivariate regression using the significant risk factors (P < 0.05) from the bivariate analysis as covariates. Results were presented as coefficients (β) and 95% confidence interval (CI).

Median LKD age was 45 years (range 23–71 years), 82% were Caucasians, and 84% were females. The median pre-donation body mass index (BMI) was 25.7 kg/m2 (range 20.4 kg/m^2^–35.7 kg/m^2^). The baseline systolic blood pressure (SBP) and diastolic blood pressure (DBP) median (and range) values were 121.5 mm Hg (98.0–151.0 mm Hg) and 76.5 mm Hg (62.0–90.0 mm Hg), respectively. The baseline pre-donation median eGFR was 95 mL/min. (range = 65–122 mL/min). The 1-year post-DN median eGFR was 63 mL/min. (range = 48–95 mL/min). The mean percentage (SD) and absolute (SD) 1-year post-DN eGFR reduction were 30.0% (SD = 8.1) and 29.9 mL/min (SD = 1.6), respectively. The 50th, 75th, 90th, and 95th eGFR reduction percentiles at 12 months post-DN corresponded to percentage (and absolute) eGFR decrements of 31.8% (30 mL/min), 36.7% (35.0 mL/min), 38.8% (40.8 mL/min), and 40.0% (46.2 mL/min), respectively (Figure 1). On OLSR and QR on the 50th percentile eGFR reduction (Figure 1; Supplementary Table S1), the following were non-significant risk factors for renal function reduction at year-one post-DN: age at donation, baseline eGFR, body mass index, systolic BP (SBP), diastolic BP (DBP), mean arterial pressure (MAP), pulse pressure (PP), body surface area (BSA), sex, and race. Baseline eGFR was a risk factor for 75th percentile eGFR reduction (Supplementary Table S1). Based on unadjusted analysis, BMI and baseline DBP were positive and negative risk factors for 90th percentile eGFR reduction; however, baseline DBP was not significant on adjusted analysis (Supplementary Table S1). Baseline BMI, DBP, eGFR, and female sex were risk factors on unadjusted and adjusted analyses for 95th percentile eGFR reduction (Figure 1; Supplementary Table S1).

**FIGURE 1:**
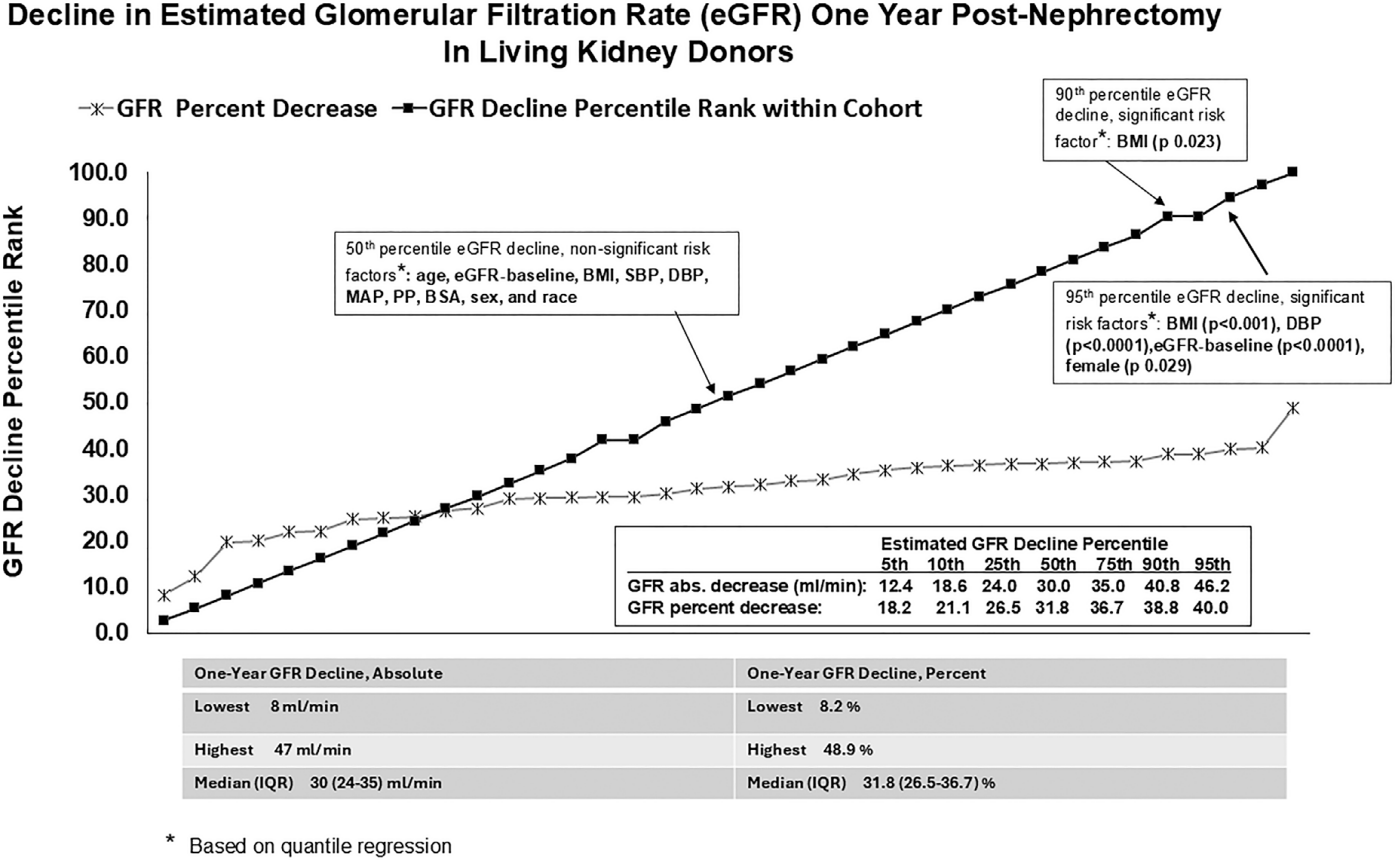
Decline in estimated glomerular filtration rate (eGFR) one year post-nephrectomy in living kidney donors.

Aside from the known detrimental outcome of obesity-associated glomerulopathy, high BMI has been associated histologically with renal tubular vacuolization and vascular hyalinosis [6]. It is associated with an unfavorable renal prognosis post-DN due to the lack of reserve capacity for compensation in the remaining kidney [3, 7]. Our study showed that the detrimental impact of high pre-donation BMI is most evident with higher severity of renal function reduction. High pre-donation BMIs were associated with eGFR reductions in the 90th and 95th percentiles (corresponding to minus 41 mL/min and 48 mL/min absolute eGFR drop) at 1-year post-DN. High pre-donation BMI was not associated with eGFR reductions in the lower (50th and 75th) percentiles.

Using central tendency-based statistics, Tent et al. [8] showed that living kidney donors with hypertension had stable renal function post-donation. Our study also showed no association between BP parameters (SBP, DBP, MAP, and PP) and the 50th, 75th, and 90th percentiles of eGFR reduction 1-year post-DN. However, baseline DBP was a risk factor, while baseline SBP was a mitigating factor, for eGFR reduction in the 95th percentile. We acknowledge that the conflicting associations of pre-DN SBP and DBP with renal function changes of LKDs seen in this study have not been reported previously and will need confirmation. However, we are aware of the report that DBP tends to be more strongly associated with CKD and renal function deterioration than SBP in the non-LKD population [9].

In a longitudinal study, Berglund et al. [10] reported a higher first-visit GFR among LDs who later experienced a reduction in post-donation GFR. In this study, we saw a similar phenomenon in the association of pre-DN eGFR with the 75th and 95th percentile eGFR reduction 1-year post-DN.

In summary, our results indicate that at 1-year post-DN, risk factors were not associated with mean or median eGFR reduction; however, pre-DN BMI is a risk factor for eGFR reduction in the 90th and 95th eGFR reduction percentiles, and pre-DN DBP, eGFR, and female sex were additional risk factors for eGFR reduction in the 95th percentile. Our study is limited by low LKD nephrectomy volume that could impact center experience and outcomes, and the small sample analyzed limited statistical power. Nevertheless, it shows that quantile regression could be used to uncover relationships between risk factors and outcome distributions beyond the mean or median in transplantation research. Our findings are hypothesis-generating and need to be confirmed by larger studies with longer follow-up, preferably using QR analysis.

## Data Availability

The original contributions presented in the study are included in the article/Supplementary Material, further inquiries can be directed to the corresponding author.
