# Supplementary material for: Risk Factors for One-Year Post-Nephrectomy Decline in Renal Function of Living Kidney Donors: Quantile Regression Analysis Based on Estimated Glomerular Filtration Rate Reduction Percentiles
Source: Transpl Int. 2025 Jun 23;38:14749. doi: 10.3389/ti.2025.14749 (PMC12229943; doi:10.3389/ti.2025.14749)
Supplement: Supplementary file 1 [file Table1.DOCX]

**SUPPLEMENTARY TABLE S1 Univariate and multivariate regression analyses for determining factors associated with percent decline in pre- and one year post-donor nephrectomy estimated glomerular filtration rate in living kidney donors.**

| **Ordinary Linear Regression** |  | Univariate Analysis |  |  |  |  |  |  |
| --- | --- | --- | --- | --- | --- | --- | --- | --- |
|  | coefficients (β) | CI-LL | CI-UL | P |  |  |  |  |
| Age | 0.109 | -0.139 | 0.357 | 0.378 |  |  |  |  |
| Baseline eGFR | 0.109 | -0.085 | 0.302 | 0.262 |  |  |  |  |
| Race, Caucasian | -3.841 | -10.702 | -10.702 | 0.263 |  |  |  |  |
| Sex, Female | 4.051 | 3.832 | 11.933 | 0.304 |  |  |  |  |
| BSA | 3.297 | -12.066 | 18.660 | 0.665 |  |  |  |  |
| BMI | -0.048 | -0.698 | 0.601 | 0.881 |  |  |  |  |
| Systolic BP | 0.035 | -0.220 | 0.291 | 0.782 |  |  |  |  |
| Diastolic BP | -0.219 | -0.610 | 0.173 | 0.264 |  |  |  |  |
| MAP | -0.094 | -0.453 | 0.265 | 0.600 |  |  |  |  |
| Pulse Pressure | 0.248 | -0.102 | 0.597 | 0.159 |  |  |  |  |
|  |  |  |  |  |  |  |  |  |
| **50th Percentile eGFR Decline -Quantile Regression** | | |  |  |  |  |  |  |
|  |  | Univariate Analysis |  |  |  |  |  |  |
|  | coefficients (β) | CI-LL | CI-UL | P |  |  |  |  |
| Age | 0.018 | -0.299 | 0.336 | 0.926 |  |  |  |  |
| Baseline GFR | 0.150 | -0.024 | 0.324 | 0.164 |  |  |  |  |
| Race, Caucasian | -3.100 | -10.004 | 3.804 | 0.465 |  |  |  |  |
| Sex, Female | 3.100 | -5.032 | 11.232 | 0.535 |  |  |  |  |
| BSA | 0.783 | -18.238 | 19.804 | 0.946 |  |  |  |  |
| BMI | -0.034 | -0.862 | 0.795 | 0.947 |  |  |  |  |
| Systolic BP | -0.012 | -0.324 | 0.300 | 0.951 |  |  |  |  |
| Diastolic BP | -0.300 | -0.731 | 0.131 | 0.260 |  |  |  |  |
| MAP | -0.036 | -0.495 | 0.422 | 0.897 |  |  |  |  |
| Pulse Pressure | 0.314 | -0.028 | 0.657 | 0.140 |  |  |  |  |
|  |  |  |  |  |  |  |  |  |
| **75th Percentile eGFR Decline -Quantile Regression** | | |  |  |  |  |  |  |
|  |  | Univariate Analysis |  |  |  |  |  |  |
|  | coefficients (β) | CI-LL | CI-UL | P |  |  |  |  |
| Age | 0.016 | -0.130 | 0.161 | 0.859 |  |  |  |  |
| **Baseline GFR** | **0.150** | **0.042** | **0.258** | **0.029** |  |  |  |  |
| Race, Caucasian | -0.300 | -4.067 | 3.467 | 0.897 |  |  |  |  |
| Sex, Female | -0.200 | -4.995 | 4.595 | 0.946 |  |  |  |  |
| BSA | 0.222 | -9.244 | 9.688 | 0.969 |  |  |  |  |
| BMI | 0.023 | -0.352 | 0.397 | 0.922 |  |  |  |  |
| Systolic BP | 0.035 | -0.093 | 0.162 | 0.658 |  |  |  |  |
| Diastolic BP | 0.025 | -0.225 | 0.275 | 0.870 |  |  |  |  |
| MAP | 0.021 | -0.196 | 0.238 | 0.872 |  |  |  |  |
| Pulse Pressure | 0.040 | -0.195 | 0.275 | 0.782 |  |  |  |  |
|  |  |  |  |  |  |  |  |  |
| **90th Percentile eGFR Decline -Quantile Regression** | | |  |  |  |  |  |  |
|  |  | Univariate Analysis |  |  |  | Multivariate  Analysis |  |  |
|  | coefficients (β) | CI-LL | CI-UL | P | coefficients (β) | CI-LL | CI-UL | P |
| Age | -0.131 | -0.274 | 0.012 | 0.141 |  |  |  |  |
| Baseline GFR | 0.100 | -0.016 | 0.216 | 0.164 |  |  |  |  |
| Race, Caucasian | -1.200 | -3.012 | 0.612 | 0.284 |  |  |  |  |
| Sex, Female | -1.600 | -9.628 | 6.428 | 0.745 |  |  |  |  |
| BSA | -0.029 | -12.561 | 12.503 | 0.997 |  |  |  |  |
| BMI | **0.642** | **0.159** | **1.126** | **0.036** | **5.259** | **1.343** | **9.174** | **0.023** |
| Systolic BP | 0.000 | -0.212 | 0.212 | >0.999 |  |  |  |  |
| Diastolic BP | **-0.200** | **-0.365** | **-0.035** | **0.049** | 1.701 | -1.793 | 5.194 | 0.051 |
| MAP | 0.000 | -0.299 | 0.299 | >0.999 |  |  |  |  |
| Pulse Pressure | 0.000 | -0.299 | 0.299 | >0.999 |  |  |  |  |
|  |  |  |  |  |  |  |  |  |
| **95th Percentile eGFR Decline -Quantile Regression** | | |  |  |  |  |  |  |
|  |  | Univariate Analysis |  |  |  | Multivariate Analysis |  |  |
|  | coefficients (β) | CI-LL | CI-UL | P | coefficients (β) | CI-LL | CI-UL | P |
| Age | -0.048 | -0.114 | 0.017 | 0.235 |  |  |  |  |
| Baseline GFR | **0.125** | **0.102** | **0.148** | **<0.0001** | **0.1903100** | **0.1903080** | **0.1903200** | **<0.0001** |
| Race, Caucasian | 0.200 | -2.609 | 3.009 | 0.907 |  |  |  |  |
| Sex, Female | **-3.000** | **-5.112** | **-0.888** | **0.025** | **1.8448000** | **1.7978690** | **1.8917320** | **0.029** |
| BSA | -0.712 | -7.904 | 6.479 | 0.872 |  |  |  |  |
| BMI | **0.899** | **0.561** | **1.237** | **<0.0001** | **1.1084300** | **1.1084250** | **1.1084350** | **<0.0001** |
| Systolic BP | **-0.075** | **-0.104** | **-0.046** | **<0.0001** | **-0.219480** | **-0.2194778** | **-0.2194761** | **<0.0001** |
| Diastolic BP | **-0.532** | **-0.640** | **-0.423** | **<0.0001** | **0.2746800** | **0.2746495** | **0.2747124** | **<0.0001** |
| MAP | **-0.348** | **-0.524** | **-0.173** | **0.002** | -0.126700 | -0.2653224 | 0.0119138 | 0.0840 |
| Pulse Pressure | -0.071 | -0.192 | 0.051 | 0.345 |  |  |  |  |

Data are regression coefficients (β) with their 95% CI that indicate the association between risk factor, and percent change CKD-EPI eGFR between pre-kidney donation and one-year post-kidney donation. Percent change in estimated glomerular filtration rate (eGFR) is the dependent variable in all models.

Glomerular filtration rate, GFR; body surface area, BSA; body mass index, BMI; blood pressure, BP; mean arterial pressure, MAP

CI-LL 95%, confidence interval lower limit

CI-UL, 95% confidence interval upper limit

P-value <0.05 indicates a statistically significant regression coefficient.
